# Healthcare providers’ perceptions of immigrant patients’ values about sexual and reproductive rights: a cross-sectional comparison with immigrants’ self-reported values

**DOI:** 10.1186/s12978-025-02161-4

**Published:** 2025-10-08

**Authors:** Andrey Tibajev, Irina Vartanova, Pontus Strimling, Birgitta Essén

**Affiliations:** 1 Department of Women’s and Children’s Health, Uppsala University, Uppsala, Sweden; 2 Institute for Futures Studies, Stockholm, Sweden

**Keywords:** Immigrants, Healthcare, Sexual and reproductive health, Culture, Stereotypes

## Abstract

**Background:**

Stereotypes about immigrant patients in healthcare can hinder effective communication, erode trust, and contribute to discrimination and poorer health outcomes. Culturally competent healthcare aims to improve communication and build trust by acknowledging patients’ cultural backgrounds, but it also risks overemphasising cultural differences and reinforcing stereotypes. To address this tension, this study examines how healthcare providers in Sweden perceive the values of immigrant patients regarding sexual and reproductive rights and compares these perceptions with the actual values held by newly arrived immigrants.

**Methods:**

The study draws on two large-scale surveys conducted in 2020–2021. The first survey was conducted among newly arrived immigrants in Sweden and included eighteen questions on moral issues related to sexual and reproductive rights, such as abortion, contraception, and sex before marriage. Responses from newly arrived immigrants originating from the Middle East, North Africa, and the Horn of Africa (*N* = 992) were used in this study. The second survey was conducted among healthcare providers working in sexual and reproductive healthcare (*N* = 1,041) and asked how they believed immigrant patients from corresponding regions would respond on the same set of issues. Results from the two surveys were compared using three complementary approaches: examining the distribution of healthcare providers’ perceptions, assessing their accuracy relative to immigrants’ self-reported values, and testing for systematic biases in the direction of these perceptions.

**Results:**

Of the eighteen included issues, healthcare providers’ perceptions aligned with immigrants’ self-reported issue positions in only six cases. The greater the proportion of immigrants holding liberal rather than conservative positions, the more often providers’ perceptions were incorrect (Pearson’s *r* = -0.73). Healthcare providers often erroneously assumed that immigrant patients hold very conservative values, a pattern most pronounced on issues where immigrants held the most liberal views.

**Conclusions:**

To avoid stereotypes and promote equitable, culturally competent healthcare, providers need to develop a better understanding of immigrants’ values and how these are shaped. Our findings reveal a tendency to underestimate the liberal values of immigrant patients, particularly on issues related to sexual and reproductive rights, thereby reinforcing bias and contributing to essentialist thinking in clinical practice.

**Supplementary Information:**

The online version contains supplementary material available at 10.1186/s12978-025-02161-4.

## Plain language summary

When people move to a new country, they often face challenges in getting the healthcare they need, especially when doctors or nurses make assumptions about their cultural or moral beliefs. In Sweden, like in many other countries, healthcare providers are encouraged to offer culturally competent care. This means they should try to understand and respect patients’ different backgrounds. However, if providers rely too much on general ideas about where someone comes from, they may end up using stereotypes instead of seeing the individual. This study looks at how healthcare providers in Sweden think about the values of immigrant patients from the Middle East, North Africa, and the Horn of Africa, specifically on issues related to sexual and reproductive rights, such as abortion, contraception, or sex before marriage. We compared what healthcare providers believe such immigrant patients think with what immigrants from these regions actually answered in two recent surveys. The results show that healthcare providers often assume that immigrant patients have overly conservative views. This mismatch was most striking on the topics where the patients were the most socially liberal. These misunderstandings can affect the quality of care that immigrant patients receive. To avoid this, healthcare workers need better knowledge of the real beliefs and experiences of their patients. This would help ensure respectful, equitable, and inclusive healthcare for everyone.

**Supplementary Information:**

The online version contains supplementary material available at 10.1186/s12978-025-02161-4.

## Background

Culture fundamentally shapes healthcare interactions. Research shows that cultural barriers can compromise the quality of care for immigrants, underscoring the importance of culturally competent practices to improve accessibility, communication, and health outcomes [1–4]. Sexual and reproductive healthcare, as well as sexual and reproductive rights (SRR), are particularly sensitive in this regard, as they are highly politicised domains in which norms and values are closely intertwined with behaviour and medical practices [5]. Values are thus intrinsically linked to interactions and decision-making in key reproductive health services, such as maternity care and contraceptive counselling.

Immigrant patients often seek healthcare in a cultural context different from the one in which their expectations and experiences were formed. Cultural norms from their origin countries shape their healthcare needs and expectations, making approaches that respect their values and avoid judgement essential [6, 7]. Studies show that when immigrant women struggle to understand or be understood by health professionals, they may experience anxiety, fear, insecurity, and discrimination, which can discourage healthcare use [2, 8, 9]. These challenges are often shaped by communication barriers, limited information, conflicting identities, and cultural taboos, and are compounded when healthcare systems fail to provide respect and understanding [1, 10, 11]. When healthcare providers misinterpret or stereotype patients’ values, diagnosis, treatment, and follow-up can be compromised, reducing the quality of SRH and widening outcome disparities [12].

Efforts to improve healthcare quality for immigrants and minority groups have increasingly focused on cultural competence, defined as the ability of providers and systems to meet the cultural needs of a diverse patient population [13, 14]. Culturally competent healthcare seeks to build trust and improve communication by enhancing providers’ understanding of cultural diversity [15]. A central premise is that care should be based on informed knowledge of patients’ values and perspectives rather than on stereotypical assumptions [13]. This is particularly important in clinical encounters characterised by power and knowledge asymmetries, such as those in sexual and reproductive healthcare [16], where subtle forms of bias can reinforce interpersonal and structural inequities [17].

Many immigrants to Western societies come from regions where norms and values related to SRR and gender relations are often more conservative than in Western contexts, such as the Middle East and North Africa (MENA) and the Horn of Africa (HoA) [18, 19]. In these regions, practices such as contraceptive use [20], abortion [21], intimate partner violence [22–24], female genital cutting [25], and hymen ‘repair’ [26] can also differ markedly from those in Western countries. Immigrants from these regions often show moderate support for SRR issues such as abortion and contraception, falling between the positions of their origin and destination countries [27–30]. However, Western narratives frequently exaggerate cultural divides, portraying immigrants from MENA and HoA as holding overly conservative and culturally incompatible values relative to the majority population [31, 32]. Overemphasising cultural differences through practices such as othering and stereotypical assumptions can lead to essentializing, perpetuating inequities, and ultimately resulting in suboptimal care [33–35].

Despite the centrality of cultural competence in improving immigrants’ healthcare experiences, few studies directly assess how culturally competent healthcare providers actually are. Research more often examines whether providers view cultural knowledge as important or consider themselves knowledgeable [14, 36]. Given the direct connection between providers’ cultural knowledge and immigrants’ experiences of care, it is crucial to ask whether healthcare providers accurately understand the culture and values of their immigrant patients. The purpose of this paper is to assess the perceptions that healthcare providers in Sweden have of immigrant patients’ values regarding SRR, and to compare these perceptions with the actual values of newly arrived immigrants to determine how well they align.

Our main contribution is combining two strands of research in reproductive health that are rarely examined together: the emphasis on cultural competence as a means to improve immigrants’ healthcare experiences and empirical analyses of immigrants’ values within SRR. This integration advances the field in several ways. First, it examines how healthcare providers perceive the values of vulnerable immigrant groups within Swedish sexual and reproductive healthcare. Second, it moves beyond self-assessments of cultural knowledge by directly measuring providers’ perceptions of immigrant patients’ values. Third, it offers a comparative benchmark by matching these perceptions with immigrants’ self-reported values, enabling an assessment of their accuracy. By comparing perceptions with actual values, we avoid assuming that healthcare providers can only hold negative stereotypes, recognising instead that perceptions may be accurate or even positively biased. As the main strategies to increase cultural competence are training and professional development [14], developing better ways to measure and evaluate providers’ cultural knowledge and value perceptions has direct implications for improving reproductive health services.

### Conceptual framework

To assess how closely healthcare providers’ perceptions align with immigrants’ values, and in what direction potential biases occur, we require a clear conceptualisation of values and their relationship to specific issue positions. In prior research, such categorisations, whether comparing countries or native-born and immigrant populations, have often been made informally based on modernisation theory [37]. This framework assumes that positions supporting sexual liberation and gender equality are liberal or modern, while opposing positions are conservative or traditional. However, this approach suffers from circular logic: issue positions are labelled liberal because they are more common in Western contexts, while the Western world is considered liberal because of these same positions. Crucially, this framing risks reinforcing divisive narratives that exaggerate differences between the West and regions such as MENA and HoA, perpetuating harmful stereotypes in academic discourse.

To avoid such circular logic and normative assumptions, we draw on the moral foundations and moral arguments theories [38, 39]. These theories link specific issue positions to broader values by analysing the moral arguments people use to justify their views. Research applying these frameworks shows that populations worldwide agree on which moral arguments align with particular positions, regardless of personal opinion, and that global differences in views arise from variations in moral argument preferences [40, 41]. This approach offers a conceptually grounded, non–Western-centric basis for comparing values across diverse populations.

#### The Swedish context

The present study is set in Sweden, where approximately 20% of the population are immigrants, with substantial cohorts from MENA and HoA arriving in recent decades [42]. The Swedish healthcare system is therefore a multicultural setting where providers and patients from diverse backgrounds interact, with a growing number of non-Western immigrants among service users.

Alongside this demographic diversity, Sweden is a global outlier in values, particularly in areas such as SRR and gender equality [18], and healthcare providers working in sexual and reproductive healthcare tend to be even more supportive of sexual liberalisation and gender egalitarianism than the general population [43]. State policies emphasise cultural competency, promoting patient-centred care and cultural relativism in recognition of different cultures and values [44, 45]. Previous studies highlight the challenges Swedish healthcare providers face in balancing their own values with multicultural considerations, especially in sexual and reproductive healthcare [46, 47]. These challenges are compounded when culture-specific assumptions about sexuality and reproduction overshadow patients’ perspectives, including, for example, in sexual and reproductive counselling [48, 49].

Immigrant women in Sweden also experience worse maternal health outcomes [50], a pattern evident across high-income countries [51], where culture and communication barriers similarly affect care [8]. The combination of these common challenges and Sweden’s explicit policy focus on culturally competent healthcare makes it a particularly well-suited setting for the present study.

## Methods

### Study design

Data come from two surveys conducted within *Migration and equity in sexual and reproductive health*, a Swedish national research environment in the field of migration and integration. The first examined the positions of immigrants, in this study defined as foreign-born individuals, on a range of SRR issues. The second surveyed healthcare providers in sexual and reproductive healthcare, asking how they perceive immigrant patients from these regions would respond to the same questions.

To assess potential biases among healthcare providers, i.e., if immigrants are perceived as overly liberal or overly conservative, we code each issue according to a general value scale using the moral foundations and moral arguments theories [39]. Following the theory, arguments associated with authority, purity, and tradition represent conservative values, whereas arguments linked to equality, freedom, and harm reduction represent liberal values.

### Data sources

The first survey, the *Swedish Immigrant Values Survey* (SIVS), collected responses from foreign-born students enrolled in Swedish for Immigrants (SFI) programs during the period of 2020–2021. SFI is Swedish language education for adults. Organised by the municipalities, SFI is a part of the mandatory public integration programme for refugees and family migrants to refugees. It is also available for almost all other immigrants with a residence permit, free of charge. It is an institutionalised point of entry into Swedish society and a well-suited site to recruit respondents at a reasonable cost, reaching a large proportion of newly arrived immigrants who are otherwise difficult to survey through more traditional means. The survey was available in Arabic, Dari, English, Somali, Swedish, and Tigrinya. It was administered digitally, both in classrooms via tablets and later, due to the pandemic, through email invitations to all SFI students in a municipality. In total, the data collection covered 37% of all 290 municipalities in Sweden, containing approximately 71% of all SFI students. The response rate for the email invitations was 6%. The SIVS contains a total of 3,871 respondents. Further details about the survey and data collection methods can be found in the documentation and codebook prepared for the survey [52].

The second survey, the *Health Care Providers’ Values and Knowledge about Sexual and Reproductive Health and Migration* (healthcare provider survey), targeted professionals working in sexual and reproductive healthcare. Respondents were recruited between January and May 2021 through their workplaces and via professional associations for Swedish gynaecologists and midwives. A digital questionnaire was made accessible to all participants without any exclusions based on workplace or profession, resulting in a total of 1,257 anonymous respondents. The sample represented approximately 30% of all gynaecologists/obstetricians and 10% of all midwives in Sweden, as compared with official statistics [43, 53]. Additional information regarding the survey and data collection methods can be found in the documentation and codebook prepared by the research group at International Maternal and Reproductive Health and Migration (IMHm) [54].

### Samples used

The analytical sample of immigrants was constructed by including only immigrants from MENA and HoA (excluding 2,674 respondents) and who are newly arrived, i.e., have been in Sweden no longer than five years (excluding an additional 205 respondents). The latter restriction was applied due to the nature of data collection through SFI, as there was a risk that individuals in SFI after such a long time in the country are outliers and may differ on unobserved characteristics that can be correlated with their values. All results using this sample are weighted by the distribution of gender, education, and region of origin among newly arrived immigrants in Sweden, to enhance the representativeness of immigrants from MENA and HoA [55].

The analytical sample of healthcare providers was constructed by excluding 216 respondents who did not work as a physician, midwife, or hospital social worker in a sexual and reproductive healthcare clinic, or were older than 67 years (the Swedish retirement age).

The number of included respondents for each issue from both surveys can be found in Table A1 in the Appendix.

Throughout the subsequent sections, we use the term *immigrants* to refer to respondents from MENA and HoA who have been in Sweden for a maximum of five years, and *healthcare providers* to refer to physicians, midwives, and hospital social workers under the age of 67 working in sexual and reproductive healthcare.

### Pilot testing

The SIVS underwent a single round of pilot testing among SFI students. The healthcare provider survey underwent two rounds of testing. First, a pilot test of the entire survey was conducted with 20 respondents from the target group, followed by qualitative interviews. The results indicated a need to revise the framing of the perception questions. After making these adjustments, we conducted a second pilot test of the revised survey instrument with 200 respondents outside the target group to assess the effectiveness of the measures.

### Variables

In the SIVS, immigrants were asked about their positions on a large number of survey questions on different moral issues, primarily concerning SRR. For this study, we selected the eighteen issues common to both the SIVS and the healthcare provider survey. Six survey questions (abortion; divorce; homosexuality; husband beat wife; sex before marriage; smacking children) are derived from the World Values Survey. These questions assess the respondent’s perception of the justifiability of these practices. Additionally, three questions followed the same structure and assessed the justifiability of issues unique to the surveys used in this study (in-vitro fertilization IVF; prostitution (selling); teenage sex). Two issues (Boy if one child; 3+ child ideal) were based on inquiries regarding ideals for the sex and quantity of children, which exist in varying formulations in several international questionnaires such as the Demographic and Health Surveys. Lastly, seven issues (female genital pricking; investigate girls’ virginity; male circumcision; parents decide sex: daughter; parents decide sex: son; sex education in schools; women’s contraception rights) were specifically crafted for these surveys due to their relevance in the context of SRR.

In the healthcare provider survey, respondents were presented with the same set of questions regarding their stance on these issues. Additionally, they were instructed to imagine a patient from either Syria, Eritrea, MENA, or HoA (the origin was randomized among respondents) and consider how such a patient would respond to the same questions they were asked.

Pilot testing highlighted that respondents found it somewhat challenging to estimate what they perceived to be other people’s values. Consequently, we simplified the task by asking about extreme positions rather than average opinions. For the abortion issue and other issues using phrasing from the World Values Survey we asked about the probability of an immigrant having answered 9 or 10 on a ten-point scale. For other issues with other phrasings and answer categories, we asked about the most liberal response option available for each issue. For example, the probability of an immigrant having answered that women have the right to use contraception regardless of reason, or being for rather than against sex education in schools. To further simplify the task, we asked about an individual patient rather than that of all immigrants or immigrant patients in general, then divided the response categories into the ranges of 0–20%, 21–40%, 41–60%, 61–80%, and 81–100%.

Using the abortion issue as an example, the question regarding issue position was formulated identically in both surveys:

For each of the following alternatives, please indicate whether you think that it can never be justified, always be justified or something in between. (Justifying something means you think it can be right.) Scale 1–10, where 1 means “Never justifiable” and 10 “Always justifiable”. Abortion.

In the healthcare provider survey, the perception question was phrased as follows:

About how likely do you think this patient would agree to the following questions almost always or always can be justified? I.e., how likely it is that the patient would answer 9 or 10 on a ten-point scale: Abortion.

The wording of all issue and perception questions can be found in Table A2 in the Appendix.

The SIVS also includes questions asking respondents which moral arguments they believe underpin a given issue position, regardless of their personal opinion (results in [30]). Using these responses, and following moral arguments theory, we define an issue position as liberal if it is more strongly associated with arguments based on equality, freedom, and harm reduction than the opposite position on the same issue. Notably, our coding decisions overlap with those made using the informal method based on modernisation theory for issues examined in both our study and previous research (e.g., 18). However, using moral arguments theory allows us to systematically code the direction of all issues, including those unique to our study, based on theory and empirical evidence, rather than relying on informal judgments about which positions are more modern or liberal.

For clarity, all issues have been coded so that higher values indicate the liberal position. Reverse-coded issues, where a higher value (the liberal position) implies disagreement with the issue/statement, are indicated with an asterisk.

### Analytical strategy

As stated in the Background, the purpose of our study is to compare healthcare providers’ perceptions with immigrants’ self-reported values. Specifically, we examine the proportion of immigrants endorsing particular positions on each issue and compare this with healthcare providers’ estimates of how common those positions are. The analysis seeks to determine whether healthcare providers’ perceptions are accurate or biased in any direction.

Our analyses proceed in three steps. First, we examine the full distribution of healthcare providers’ responses to assess the variability of their perceptions and determine whether the modal perception aligns with immigrants’ actual responses. Second, we calculate the correlation between immigrants’ average issue positions and the proportion of healthcare providers whose perceptions are accurate. Third, we summarise perceptions into single-point estimates, assuming each response category of perceptions can be represented by its midpoint. We then correlate these estimates with immigrants’ issue positions to assess the existence and direction of bias and whether it is systematically related to immigrants’ values.

## Results

### Descriptive statistics

The immigrant sample comprises 992 individuals (829 from MENA and 163 from HoA). All analyses use weighted data to reflect the demographic composition of the relevant immigrant population, which includes 50% women and 19% with a university education.

The healthcare provider sample comprises 1,041 respondents (411 physicians, 594 midwives, and 36 hospital social workers). Of these, approximately 90% are women and 90% were born in Sweden. According to the best available statistics, the gender distribution is representative of the target population, although foreign-born individuals are slightly underrepresented [43].

The proportion of immigrants holding a liberal position varies considerably across the included issues (Table 1). At one end of the spectrum, only 4% consider prostitution (selling) always or almost always justifiable, and only 7% hold the same view regarding teenage sex. At the other end, 97% reject the idea that it is always or almost always justifiable for a man to beat his wife, and 96% oppose female genital pricking. On average, 54% of immigrants hold a liberal position on the included issues.

For comparison, Table 1 also presents the proportion of healthcare providers holding a liberal position on these issues.

**Table 1:** Proportion with liberal issue position among immigrants and healthcare providers

	Immigrants	Healthcare providers
Abortion	0.26	0.90
Boy if one child*	0.95	0.99
Divorce	0.44	0.84
Female genital pricking*	0.96	0.99
Homosexuality	0.28	0.96
Husband beat wife*	0.97	0.99
Investigate girls’ virginity*	0.91	1.00
IVF	0.56	0.48
Male circumcision*	0.27	0.89
Parents decide sex: daughter*	0.52	1.00
Parents decide sex: son*	0.54	1.00
Prostitution (selling)	0.04	0.03
Sex before marriage	0.30	0.89
Sex education in schools	0.74	1.00
Smacking children*	0.96	0.99
Teenage sex	0.07	0.64
Women’s contraception rights	0.54	1.00
3 + children ideal*	0.37	0.50

All issues coded 0/1, with 1 being the liberal position. Immigrants’ proportions weighted. Issues marked with * are reversed coded, i.e., that the liberal position is to disagree with the statement. See table A1 in the Appendix for phrasings

### Distribution of healthcare providers’ perceptions of immigrant patients

**Fig. 1:**
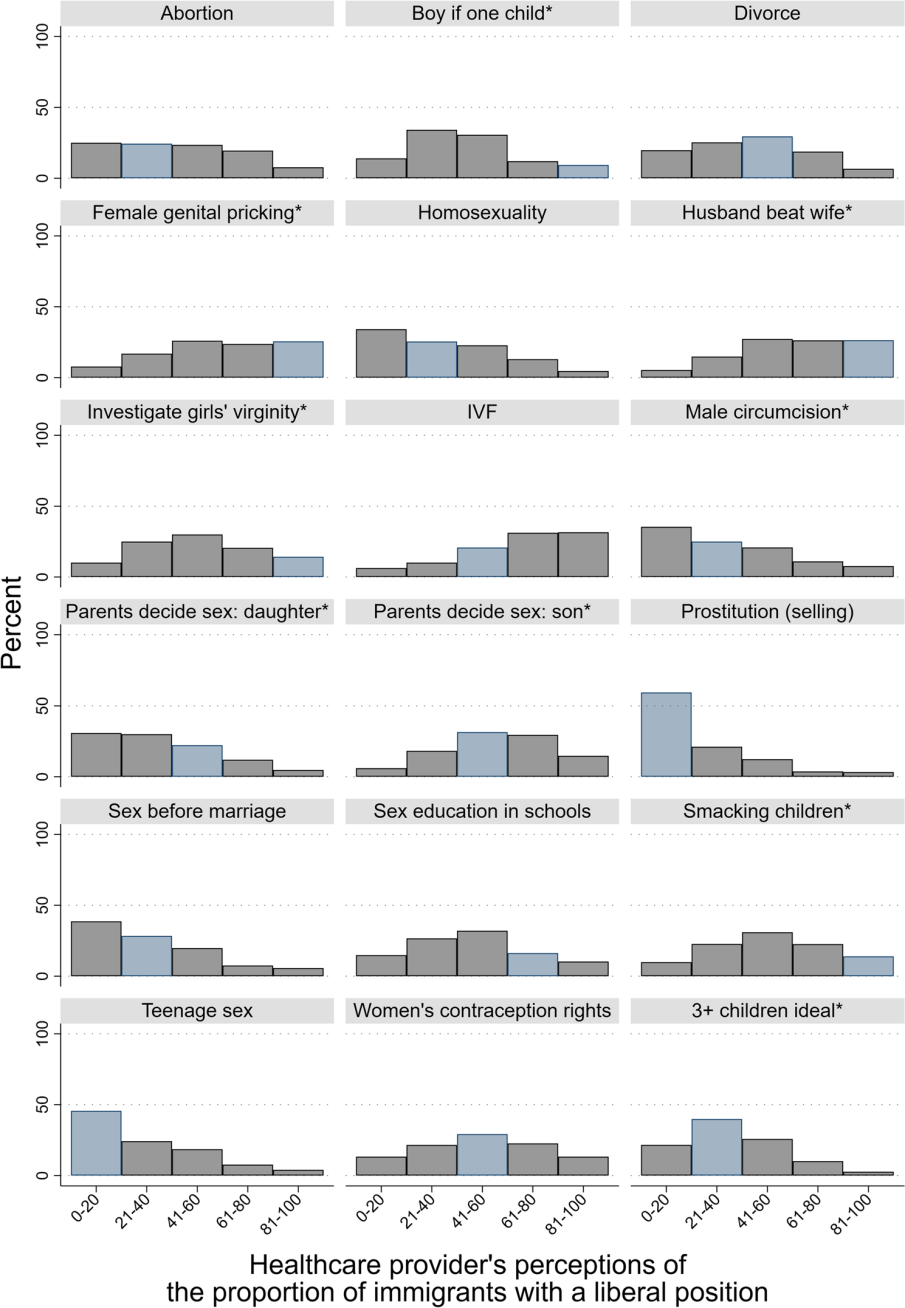
Distribution of healthcare providers’ perceptions about the values of immigrants. Blue bars indicate the correct perception

The first step examines the full range of healthcare providers’ responses to assess the distribution of perceptions. Figure 1 displays these distributions for all included issues. On the X-axis are the five answer categories for the perception questions, and on the Y-axis is the percentage of healthcare provider respondents selecting each category. The blue bars indicate the category that matches the actual positions of immigrants. For example, 44% of immigrants indicated that divorce is always or almost always justifiable (see Table 1). The plurality of healthcare providers correctly perceived this, as shown by the middle blue bar.

However, across all eighteen issues, the modal perception was correct in only six cases. For one issue (IVF), the modal perception was too liberal, while for eleven issues, it was too conservative. For instance, almost no immigrant agreed that investigating girls’ virginity is acceptable, yet the modal response among healthcare providers was that between 40 and 60% of immigrants would find it acceptable. Overall, healthcare providers selected the correct category 27% of the time, a too liberal category 27% of the time, and a too conservative category 46% of the time. These findings suggest a conservative bias in healthcare providers’ perceptions of immigrants’ values, a pattern that is explored further in subsequent analyses.

### Correlation between perception accuracy and immigrants’ values

**Fig. 2:**
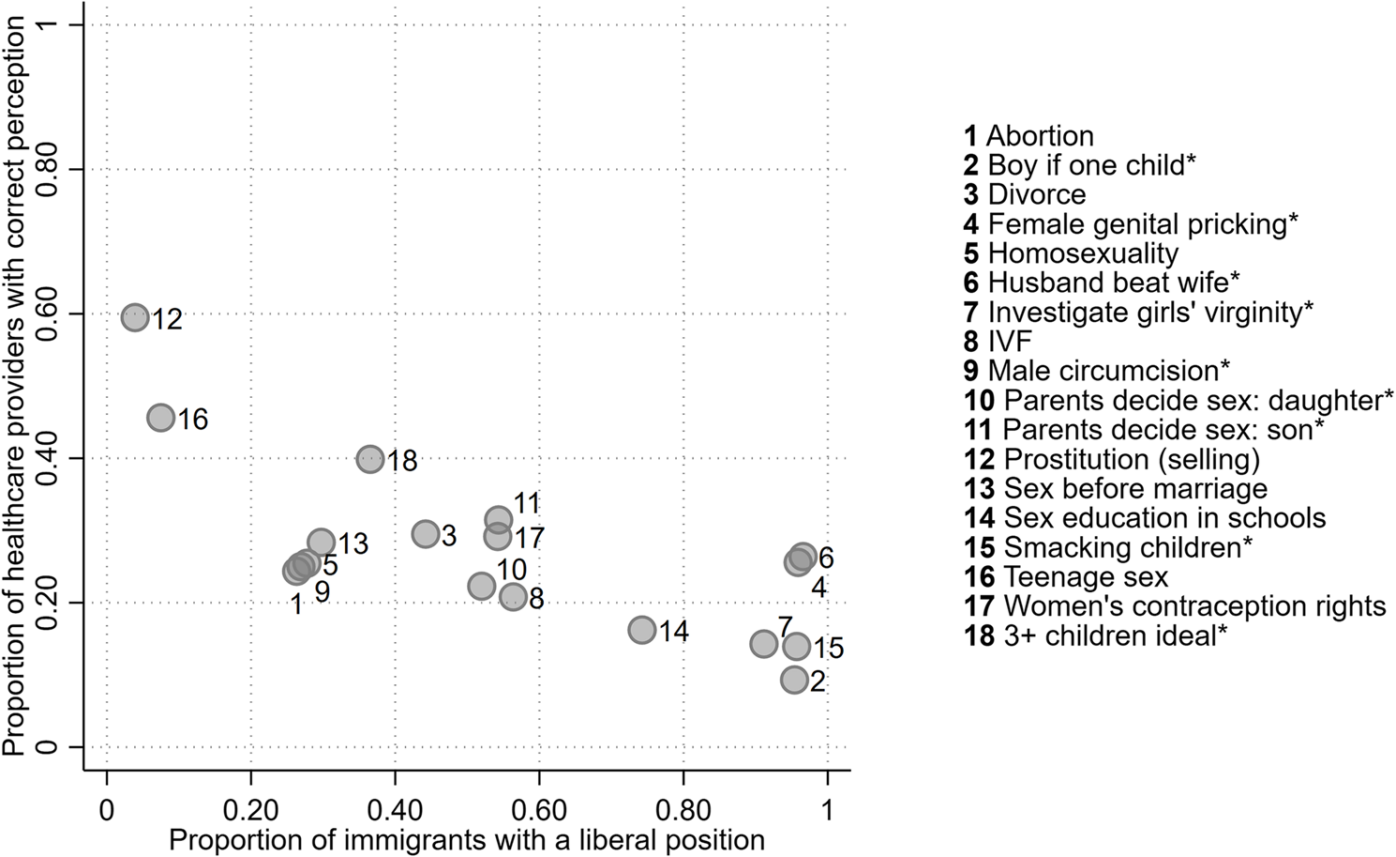
Healthcare providers’ correct perceptions and immigrants’ issue positions

In the second step, we test whether healthcare providers’ ability to correctly perceive immigrants’ positions on the issues is associated with the values held by immigrants. This is done by correlating how often healthcare providers answered correctly with the proportion of immigrants holding a liberal position on each included issue. Figure 2 displays the percentage of correct responses by healthcare providers on the Y-axis and the proportion of immigrants with a liberal position on the X-axis. The issues with the highest percentage of correct perceptions are prostitution (selling) and teenage sex. These are issues on which the lowest proportion of immigrants hold the liberal position of finding these practices justifiable. Conversely, the lowest percentage with a correct perception was observed on issues where immigrants were most likely to hold liberal opinions. This included the positions of not preferring a boy if one child and not finding smacking children to be always or almost always justifiable.

Across all issues, we find a strong negative correlation (Pearson’s *r* = −0.73) between the proportion of healthcare providers with correct perceptions and the proportion of immigrants with a liberal position. In other words, the more common it is for immigrants to hold a liberal position on an issue, the less accurate healthcare providers’ perceptions tend to be.

### Direction of bias in healthcare providers’ perceptions

**Fig. 3:**
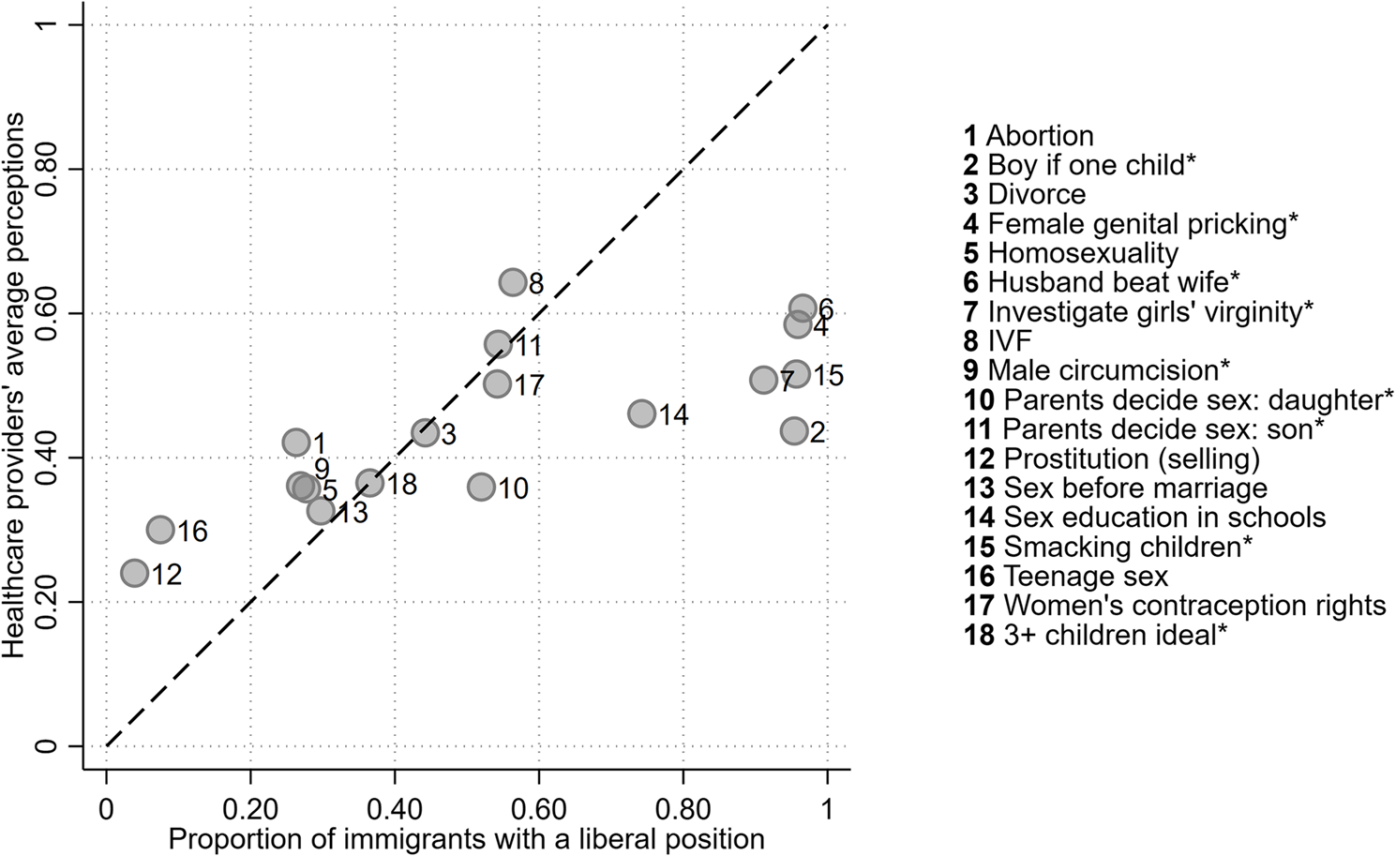
Healthcare providers’ average perceptions about the values of immigrants

In the third step, we analyse whether the direction of potential bias is correlated with immigrants’ values. We summarise healthcare providers’ responses into single-point estimates to reveal their average perceptions of immigrants’ issue positions. Figure 3 displays these point estimates on the Y-axis versus the proportion of immigrants with a liberal position on the X-axis. The dashed 45-degree line represents alignment between healthcare providers’ perceptions and immigrants’ actual positions, indicating that, on average, perceptions were correct for a given issue.

Most issues lie on or close to the dashed 45-degree line, suggesting that despite variability in perceptions among healthcare providers, their average perceptions are correct for issues such as divorce, male circumcision, homosexuality, sex before marriage, and women’s contraception rights. This indicates that overestimations of liberal positions by some healthcare providers and underestimations by others tend to cancel each other out on these issues.

However, there is also a clear conservative bias in the average perceptions of immigrants’ positions on issues where almost all of them hold the liberal position. Specifically, nearly all immigrants take a negative stance on the preference for a boy if one child, the practices of investigating girls’ virginity, and female genital pricking, and if it is justifiable for a husband to beat his wife or parents smacking children. In contrast, healthcare providers, on average, perceived that only about half of the immigrant population would hold these positions on the five issues. These discrepancies reveal a substantial conservative bias in healthcare providers’ perceptions compared to the actual positions among immigrants from MENA and HoA.

### Additional analyses and robustness of results

Our combined results indicate that healthcare providers have a conservative bias in their perceptions of immigrants’ values on SRR, particularly for issues where almost all immigrants hold a liberal position. In this section, we present additional analyses to test the robustness of our findings, specifically, the validity of our method for measuring perceptions and the informative value of our dichotomous definition of conservative versus liberal positions on the included issues.

The task of considering others’ moral stances in a survey context may feel artificial and cognitively demanding, potentially leading to responses disconnected from reality. Thus, claims about bias among healthcare providers against immigrants depend on validating the survey instrument. To test this, a subset of respondents from the healthcare provider survey was asked to assess the moral positions of their closest peers rather than immigrant patients. These respondents provided perceptions about individuals within the same profession working in sexual and reproductive healthcare. We assume that professionals have a reasonable understanding of their colleagues’ moral positions on SRR-related issues. In other words, we assume that healthcare providers accurately perceive the values of their peers. We use this as a test of whether the survey instrument can validly measure perceptions. If the method fails this test, we cannot conclude that misaligned perceptions of immigrants’ issue positions are due to bias among healthcare providers.

Figures A1, A2, and A3 in the Appendix display the results. Overall, respondents accurately perceived their peers’ positions 71% of the time, with the modal perception corresponding to the correct issue position for sixteen of the eighteen issues. Healthcare providers struggled most with correctly perceiving positions on issues their peers were evenly divided between, rather than on any extreme end. Importantly, there was no systematic bias in perceptions toward either the conservative or liberal direction, except for an overestimation of liberal positions on IVF. In summary, healthcare providers demonstrated substantial accuracy when perceiving their peers’ positions, supporting the validity of our survey instrument.

To ease the cognitive burden on healthcare providers completing the survey, we operationalised liberal and conservative positions dichotomously, focusing on the proportion of respondents holding each position. A potential issue with this approach is that it does not account for the entire distribution of immigrants’ responses on the issues originally measured using a ten-point scale. However, Figure A4 in the Appendix demonstrates a near-perfect correlation (Pearson’s *r* = 0.96) between the average immigrant position, considering the full distribution of responses, and the proportion classified as holding a liberal position under our dichotomous operationalisation. This indicates that our results are not contingent on the dichotomous approach.

## Discussion

Culturally competent healthcare depends on providers accurately perceiving and addressing the values of immigrant patients. Our findings reveal notable discrepancies between these perceptions and the actual positions of immigrants. On the majority of included issues, the most common perception was that immigrants are more conservative than they actually are. The higher the proportion of immigrants holding a liberal rather than conservative position on an issue, the more often healthcare providers’ perceptions were incorrect. Notably, the largest conservative bias was observed on issues where almost all immigrants hold liberal positions. These include not finding it justifiable for a man to beat his wife or for parents to smack children, rejecting practices like investigating girls’ virginity or female genital pricking, and not preferring a boy if having only one child. In these cases, healthcare providers believed that around half of immigrant patients would support these positions, despite the near-universal rejection of them in the immigrant sample.

These inaccurate perceptions can have significant consequences at the level of clinical care. They are particularly relevant in sexual and reproductive healthcare, where practices are intrinsically connected with values, and provider assumptions can therefore have a direct impact on communication, trust, and decision-making. From the perspective of individual immigrant patients from these origins, our results reveal a considerable risk of being misjudged by healthcare providers who ascribe overly conservative values to them.

Such misjudgements may be compounded by healthcare practices and professional cultures that emphasise cultural differences without sufficient safeguards against essentialist thinking. Interpersonal biases are embedded within broader systems that can uphold inequities through routine provider–patient interactions [56]. These structural dynamics are especially concerning given the hierarchical power healthcare providers hold over patients in clinical settings. As Hamed and Bradby [57] argue, such practices risk perpetuating racialisation under the guise of cultural sensitivity, whereby minority patients are viewed as incompetent or excluded from decision-making processes. This conservative bias may not only contribute to inequities in care but also foster feelings of moral superiority toward immigrants, reinforcing negative attitudes and hostility [58, 59].

Our study cannot determine why healthcare providers hold the perceptions they do, but one possible explanation is that these are anchored in perceived values of immigrants’ countries of origin. However, immigrants’ values are not always representative of those in their countries of origin. Migrants often differ in opinions from those who remain behind [60, 61], and over time, their opinions become more aligned with the destination country through acculturation processes [62, 63]. In their studies of attitudes toward female genital cutting among the Somali population in Sweden, Johnsdotter and Essén [64], and Wahlberg and Essén [65] show rapid changes linked to the new material, religious, and social conditions in Sweden, prompting a reconsideration of cultural practices and attitudes. These dynamics underscore that assumptions based on static notions of origin-country values are likely to misrepresent the perspectives of current immigrant patients.

Perceptions of origin-country values can themselves be inaccurate, and using the World Values Survey [66], we can further contextualise our findings by comparing the most biased perceptions to opinions in the relevant countries of origin. Among the surveyed countries in MENA and HoA, less than 5% of the population finds it justifiable for a man to beat his wife or for parents to smack children. Globally, no country reports proportions exceeding 16% and 30% for wife-beating and child-smacking, respectively. These global patterns suggest that healthcare providers’ perceptions are not only inaccurate in the Swedish context but also misaligned even with the most conservative norms in immigrants’ countries of origin.

Our findings highlight the need for healthcare systems to recognise that these immigrant groups are often less culturally distant and conservative than commonly assumed. Increasing cultural knowledge through training, as frequently advocated in the cultural competence literature, is a useful starting point. We also echo the recommendation of a recent qualitative study of Muslim women’s experiences of contraceptive counselling in Sweden, which calls for engaging with each patient as an individual rather than relying on generalisations, and to “focus more on health system changes, including bias training for healthcare providers to achieve person-centred contraceptive services” [67].

### Strengths and limitations

This study employed a novel approach to analyse healthcare providers’ perceptions of immigrant patients from MENA and HoA regions within sexual and reproductive healthcare by directly comparing two surveys: one among newly arrived immigrants in Sweden, which included questions on moral issues related to SRR, and another among healthcare providers working in sexual and reproductive healthcare, which asked how they believed comparable immigrant patients would respond to the same set of issues. This direct test of providers’ knowledge, allowing for the possibility of both liberal and conservative biases, offers an important and innovative contribution to research on cultural competence and interactions between providers and immigrant patients.

One potential limitation is social desirability bias in immigrants’ survey responses. While this cannot be ruled out, prior analyses of the same sample found alignment with other data on immigrants in Sweden collected using probability sampling methods to ensure representativeness [30].

Another limitation is the partial mismatch between the immigrant population assumed in healthcare providers’ perceptions and the characteristics of our immigrant sample. While our sample consists of newly arrived individuals, providers were asked to reflect on immigrants from these regions more broadly. This mismatch could affect comparisons, although the large observed gaps between providers’ perceptions and immigrant respondents’ values suggest the impact is likely limited. In the survey, providers also indicated the type of immigrant they had in mind (gender, age, and length of residence in Sweden), with details provided in Table A3 in the Appendix. We also note that individuals accessing sexual and reproductive healthcare may not be fully representative of the broader immigrant population, or of any population group.

## Conclusion

Culturally competent healthcare requires that providers neither ignore nor exaggerate cultural differences, yet few studies have meaningfully assessed the accuracy of providers’ cultural knowledge. This study addresses that gap by examining how well healthcare providers’ perceptions of immigrant patients’ values align with those patients’ self-reported views. Focusing on sexual and reproductive healthcare, an area in which cultural values are closely intertwined with medical practice, we compared healthcare providers’ perceptions of immigrant patients’ SRR values with immigrants’ own self-reported values. Using two large-scale matched surveys, our analysis reveals a marked underestimation of liberal values among immigrants from MENA and HoA, underscoring the importance of engaging with patients as individuals rather than relying on assumptions.

This study demonstrates that healthcare providers commonly underestimate the liberal values held by immigrants from MENA and HoA, highlighting the critical need to move beyond stereotypes for truly culturally competent care. Future research should expand to diverse populations and employ innovative methodologies, such as randomised vignettes, to better capture and compare providers’ perceptions with patient realities, while also strengthening bias-awareness components in cultural competence training. Ultimately, enhancing our understanding of these dynamics is essential for fostering equity, trust, and respectful, person-centred care in increasingly multicultural healthcare environments.

## Supplementary Information

Supplementary Material 1.

## Data Availability

The data that support the findings of this study are available on request. The data are not publicly available due to restrictions set by the Swedish Ethical Review Authority. Requests for the The Swedish Immigrant Values Survey (doi: 10.17605/OSF.IO/9SQTN) can be sent to Pontus Strimling, pontus.strimling@iffs.se, and be made available after ethical vetting. Requests for the survey on Health Care Providers’ Values and Knowledge about Sexual and Reproductive Health and Migration (doi: 10.17605/OSF.IO/MS9D2) can be sent to Birgitta Essén, birgitta.essen@kbh.uu.se.

## Abbreviations

SRR: Sexual and Reproductive Rights
MENA: Middle East and North Africa
HoA: Horn of Africa
SIVS: Swedish for Immigrants
IVF: In-Vitro Fertilization
