# Supplementary material for: Healthcare providers’ perceptions of immigrant patients’ values about sexual and reproductive rights: a cross-sectional comparison with immigrants’ self-reported values
Source: Reprod Health. 2025 Oct 8;22:191. doi: 10.1186/s12978-025-02161-4 (PMC12509363; doi:10.1186/s12978-025-02161-4)

Appendix:

Healthcare Providers’ Perceptions of Immigrant Patients’ Values about Sexual and Reproductive Rights: A Cross-Sectional Comparison with Immigrants’ Self-Reported Values

[**Table A1** Number of respondents across issue positions and perceptions 2](#_Toc205805776)

[**Table A2** Issues and survey questions 3](#_Toc205805777)

[**Table A3** Proportion healthcare providers who had the following characteristics of immigrants in mind when answering the perception questions 7](#_Toc205805778)

[**Figure A1** Distribution of healthcare providers perceptions about other the values of other healthcare 8](#_Toc205805779)

[providers. Blue bars indicate the correct perception. 8](#_Toc205805780)

[**Figure A2** Healthcare providers correct perceptions and healthcare providers issue positions 9](#_Toc205805781)

[**Figure A3** Healthcare providers average perceptions about the values of other healthcare providers. 10](#_Toc205805782)

[**Figure A4** Proportion of immigrants with a liberal position versus average immigrant issue position, 11](#_Toc205805783)

**Table A1** Number of respondents across issue positions and perceptions

|  | **Issue positions of** | | **Perceptions about** | |
| --- | --- | --- | --- | --- |
|  | Immigrants | Healthcare providers | Immigrants | Healthcare providers |
| Abortion | 795 | 905 | 817 | 37 |
| Boy if one child* | 799 | 906 | 709 | 29 |
| Divorce | 798 | 908 | 810 | 37 |
| Female genital pricking* | 761 | 903 | 692 | 30 |
| Homosexuality | 803 | 909 | 817 | 37 |
| Husband beat wife* | 813 | 909 | 811 | 37 |
| Investigate girls' virginity* | 780 | 904 | 700 | 30 |
| IVF | 790 | 908 | 827 | 37 |
| Male circumcision* | 750 | 898 | 700 | 30 |
| Parents decide sex: daughter* | 788 | 903 | 709 | 30 |
| Parents decide sex: son* | 786 | 903 | 712 | 30 |
| Prostitution (selling) | 798 | 903 | 807 | 37 |
| Sex before marriage | 785 | 908 | 804 | 37 |
| Sex education in schools | 771 | 905 | 709 | 30 |
| Smacking children* | 808 | 911 | 811 | 37 |
| Teenage sex | 790 | 903 | 809 | 36 |
| Women's contraception rights | 825 | 906 | 706 | 30 |
| 3+ children ideal* | 785 | 821 | 710 | 30 |

*Reversed coded in analyses, a higher value means to disagree with statement.

**Table A2** Issues and survey questions

| **Issue** | **Corresponding survey questions** |
| --- | --- |
| Abortion | Issue question in the SIVS and the healthcare provider survey:  For each of the following alternatives, please indicate whether you think that it can never be justified, always be justified or something in between. (Justifying something means you think it can be right.) Scale 1-10, where 1 means "Never justifiable" and 10 "Always justifiable". Abortion  Perception question in the healthcare provider survey:  About how likely do you think this patient would agree to the following questions almost always or always can be justified? I.e., how likely it is that the patient would answer 9 or 10 on a ten-point scale. Abortion |
| Boy if one child* | Issue question in the SIVS and the healthcare provider survey:  If you were just having one child, would you rather have a boy or a girl? [Boy] [Girl] [Does not matter]  Perception question in the healthcare provider survey:  About how likely do you think it is that the patient will answer that if they were to have only one child, that a boy is preferable to the alternatives of a girl or that it does not matter? |
| Divorce | Issue question in the SIVS and the healthcare provider survey:  For each of the following alternatives, please indicate whether you think that it can never be justified, always be justified or something in between. (Justifying something means you think it can be right.) Scale 1-10, where 1 means "Never justifiable" and 10 "Always justifiable". Divorce  Perception question in the healthcare provider survey:  About how likely do you think this patient would agree to the following questions almost always or always can be justified? I.e., how likely it is that the patient would answer 9 or 10 on a ten-point scale. Divorce |
| Female genital pricking* | Issue question in the SIVS and the healthcare provider survey:  Below are some non-medical practices that are performed on children and young people in certain cultures. How acceptable do you think the following practices are? Symbolic branding of the genital skin of girls, without removing tissue. [Acceptable] [Unacceptable] [I am not familiar with this practise]  Perception question in the healthcare provider survey:  About how likely do you think the patient would respond that non-medical practices performed on children and young people in some cultures are acceptable? Symbolic pricking/marking of genital skin on girls without tissue is removed or suturing is done. |
| Homosexuality | Issue question in the SIVS and the healthcare provider survey:  For each of the following alternatives, please indicate whether you think that it can never be justified, always be justified or something in between. (Justifying something means you think it can be right.) Scale 1-10, where 1 means "Never justifiable" and 10 "Always justifiable". Homosexuality  Perception question in the healthcare provider survey:  About how likely do you think this patient would agree to the following questions almost always or always can be justified? I.e., how likely it is that the patient would answer 9 or 10 on a ten-point scale. Homosexuality |
| Husband beat wife* | Issue question in the SIVS and the healthcare provider survey:  For each of the following alternatives, please indicate whether you think that it can never be justified, always be justified or something in between. (Justifying something means you think it can be right.) Scale 1-10, where 1 means "Never justifiable" and 10 "Always justifiable". For a man to beat his wife  Perception question in the healthcare provider survey:  About how likely do you think this patient would agree to the following questions almost always or always can be justified? I.e., how likely it is that the patient would answer 9 or 10 on a ten-point scale. For a man to beat his wife |
| Investigate girls' virginity* | Issue question in the SIVS and the healthcare provider survey:  Below are some non-medical practices that are performed on children and young people in certain cultures. How acceptable do you think the following practices are? To investigate and determine a girl’s/woman’s virginity. [Acceptable] [Unacceptable] [I am not familiar with this practise]  Perception question in the healthcare provider survey:  About how likely do you think the patient would respond that non-medical practices performed on children and young people in some cultures are acceptable? To investigate and determine the virginity of a girl/woman |
| IVF | Issue question in the SIVS and the healthcare provider survey:  For each of the following alternatives, please indicate whether you think that it can never be justified, always be justified or something in between. (Justifying something means you think it can be right.) Scale 1-10, where 1 means "Never justifiable" and 10 "Always justifiable". Assisted fertilization or in vitro fertilization (IVF)  Perception question in the healthcare provider survey:  About how likely do you think this patient would agree to the following questions almost always or always can be justified? I.e., how likely it is that the patient would answer 9 or 10 on a ten-point scale. Assisted fertilization or in vitro fertilization (IVF) |
| Male circumcision* | Issue question in the SIVS and the healthcare provider survey:  Below are some non-medical practices that are performed on children and young people in certain cultures. How acceptable do you think the following practices are? Circumcision of boys where the foreskin is removed. [Acceptable] [Unacceptable] [I am not familiar with this practise]  Perception question in the healthcare provider survey:  About how likely do you think the patient would respond that non-medical practices performed on children and young people in some cultures are acceptable? Circumcision of the foreskin of boys where tissue is removed and suturing is done. |
| Parents decide sex daughter* | Issue question in the SIVS and the healthcare provider survey:  Should the parents decide that their daughter wait with sex until she is married? [Yes] [No]  Perception question in the healthcare provider survey:  About how likely do you think it is that the patient answers that the parents should be able to decide that their daughter should wait to have sex until she gets married? |
| Parents decide sex son* | Issue question in the SIVS and the healthcare provider survey:  Should the parents decide that their son wait with sex until he is married? [Yes] [No]  Perception question in the healthcare provider survey:  About how likely do you think it is that the patient answers that the parents should be able to decide that their son should wait to have sex until he gets married? |
| Prostitution (selling) | Issue question in the SIVS and the healthcare provider survey:  For each of the following alternatives, please indicate whether you think that it can never be justified, always be justified or something in between. (Justifying something means you think it can be right.) Scale 1-10, where 1 means "Never justifiable" and 10 "Always justifiable". Prostitution, to sell one’s body for money  Perception question in the healthcare provider survey:  About how likely do you think this patient would agree to the following questions almost always or always can be justified? I.e., how likely it is that the patient would answer 9 or 10 on a ten-point scale. Prostitution, to sell one’s body for money |
| Sex before marriage | Issue question in the SIVS and the healthcare provider survey:  For each of the following alternatives, please indicate whether you think that it can never be justified, always be justified or something in between. (Justifying something means you think it can be right.) Scale 1-10, where 1 means "Never justifiable" and 10 "Always justifiable". Having casual sex before marriage  Perception question in the healthcare provider survey:  About how likely do you think this patient would agree to the following questions almost always or always can be justified? I.e., how likely it is that the patient would answer 9 or 10 on a ten-point scale. Prostitution, to sell one’s body for money |
| Smacking children* | Issue question in the SIVS and the healthcare provider survey:  For each of the following alternatives, please indicate whether you think that it can never be justified, always be justified or something in between. (Justifying something means you think it can be right.) Scale 1-10, where 1 means "Never justifiable" and 10 "Always justifiable". Parents smacking their children  Perception question in the healthcare provider survey:  About how likely do you think this patient would agree to the following questions almost always or always can be justified? I.e., how likely it is that the patient would answer 9 or 10 on a ten-point scale. Parents smacking their children |
| Sex education in schools | Issue question in the SIVS and the healthcare provider survey:  Are you for or against sex education in schools? [For] [Against]  Perception question in the healthcare provider survey:  About how likely do you think it is that the patient answers that it is that the patient answers that it is for sex education at school? |
| Teenage sex | Issue question in the SIVS and the healthcare provider survey:  For each of the following alternatives, please indicate whether you think that it can never be justified, always be justified or something in between. (Justifying something means you think it can be right.) Scale 1-10, where 1 means "Never justifiable" and 10 "Always justifiable". Underage teenagers having casual sex  Perception question in the healthcare provider survey:  About how likely do you think this patient would agree to the following questions almost always or always can be justified? I.e., how likely it is that the patient would answer 9 or 10 on a ten-point scale. Underage teenagers having casual sex |
| Women’s contraception rights | Issue question in the SIVS and the healthcare provider survey:  Do you think a woman should have the right to use contraception? [Yes, regardless of the reason] [Yes, but only if she is over 18] [Yes, but only if she is married] [Yes, but only if her husband agrees] [No, never]  Perception question in the healthcare provider survey:  About how likely do you think the patient would answer the following about a woman's right to use contraception? A woman should always have the right to use contraception, regardless of the reason. |
| 3+ child ideal* | Issue question in the SIVS and the healthcare provider survey:  How many children are ideal in a family?  Perception question in the healthcare provider survey:  About how likely do you think it is that the patient answers three or more children to the question about the ideal number of children for a family? |

*Reversed coded in analyses, a higher value means to disagree with statement.

Notes: Perception question in the healthcare provider survey translated here from the original Swedish.

**Table A3** Proportion healthcare providers who had the following characteristics of immigrants in mind when answering the perception questions

|  |  |
| --- | --- |
| Gender |  |
| Women | 78% |
| Men | 20% |
| Couples | 28% |
|  |  |
| Age |  |
| Younger | 60% |
| Middle-aged/older | 69% |
|  |  |
| Length of residence in Sweden |  |
| Newly arrived | 82% |
| In Sweden for some time | 49% |

Note: Multiple answers possible.

**Figure A1** Distribution of healthcare providers perceptions about other the values of other healthcare

providers. Blue bars indicate the correct perception.


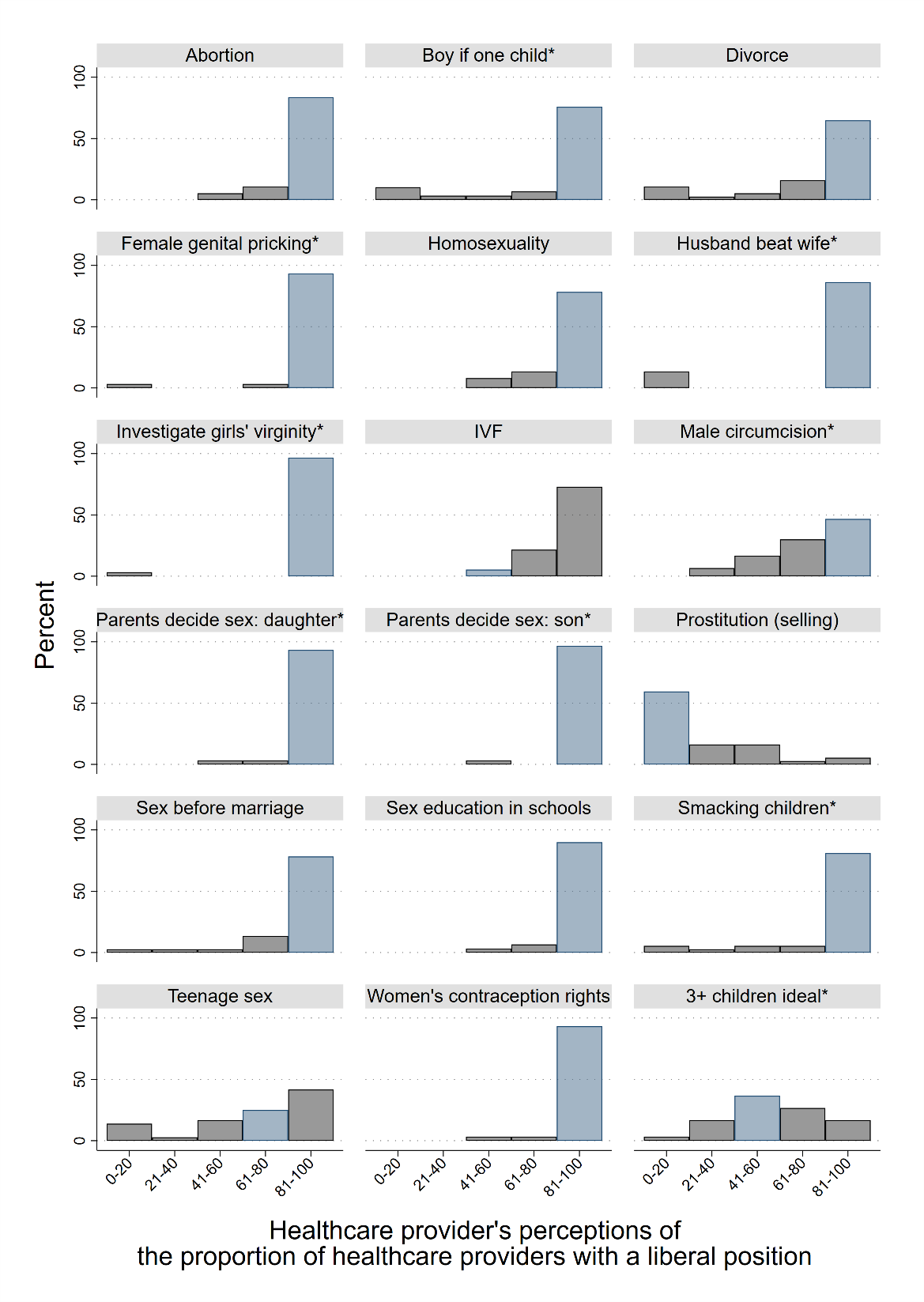


**Figure A2** Healthcare providers correct perceptions and healthcare providers issue positions


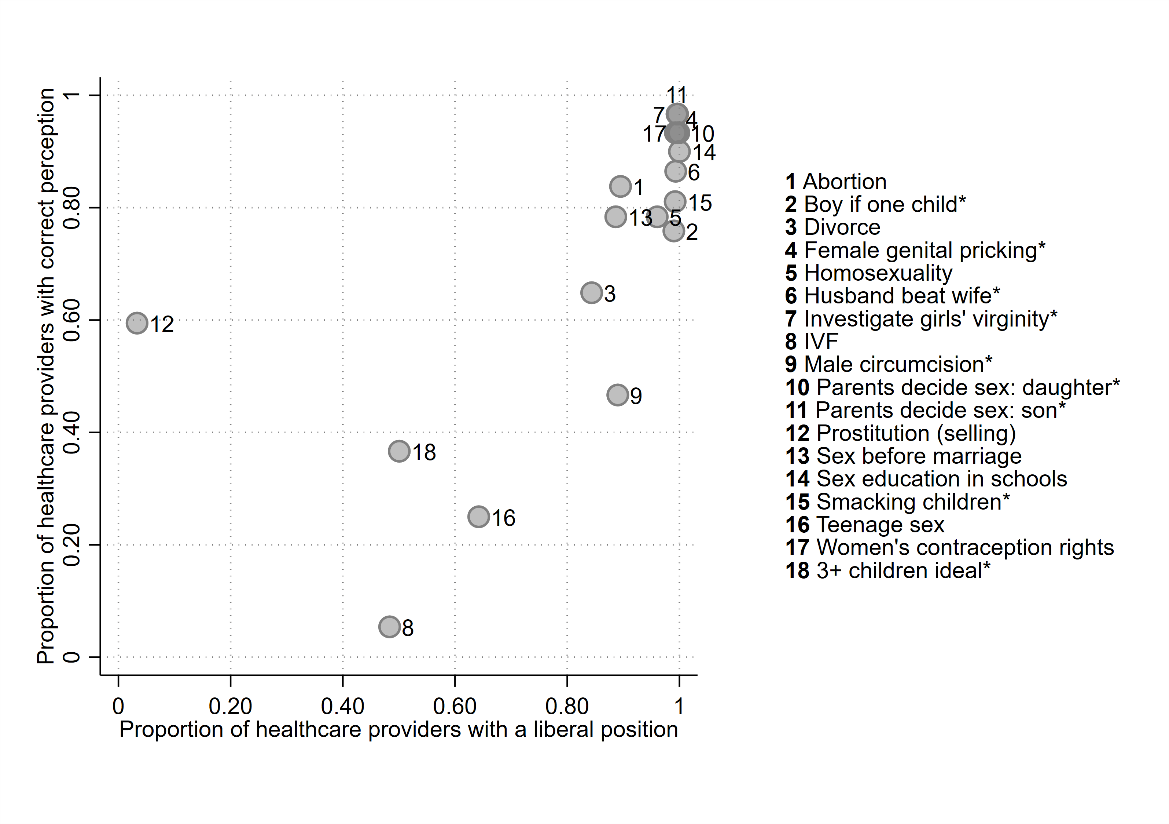


**Figure A3** Healthcare providers average perceptions about the values of other healthcare providers.


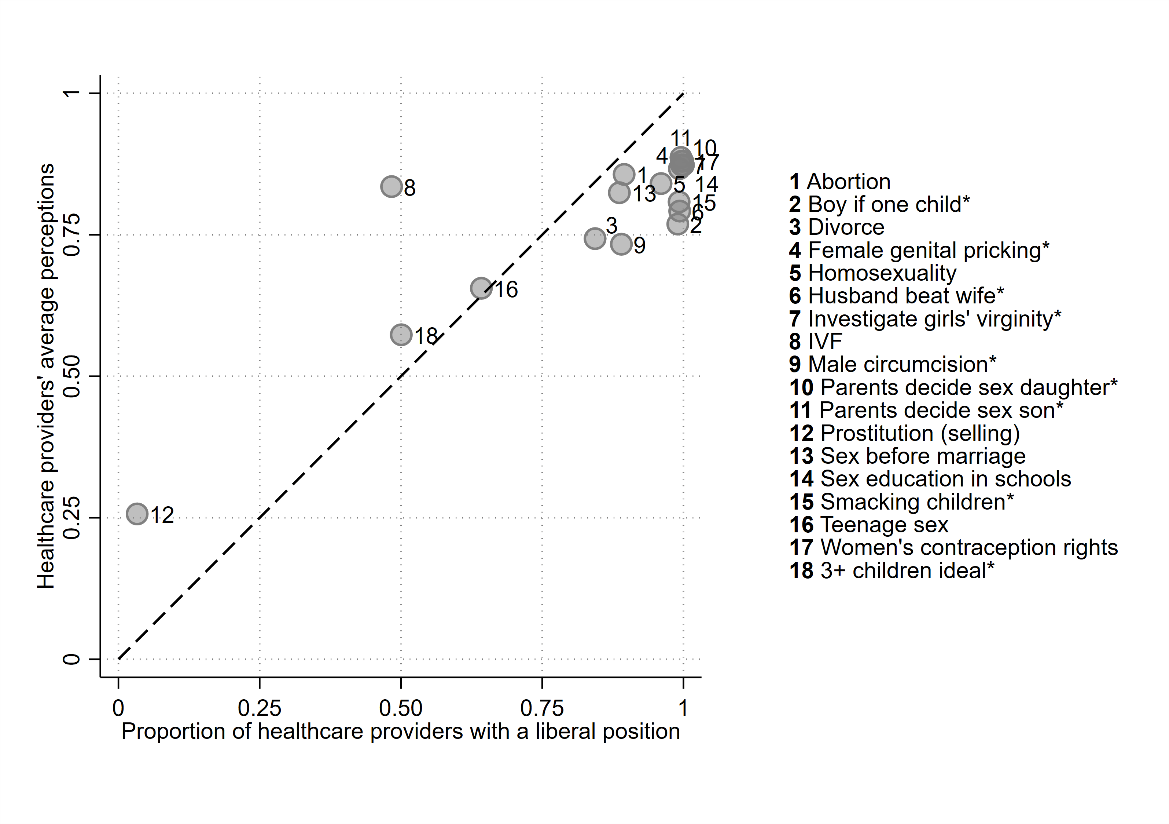


**Figure A4** Proportion of immigrants with a liberal position versus average immigrant issue position,


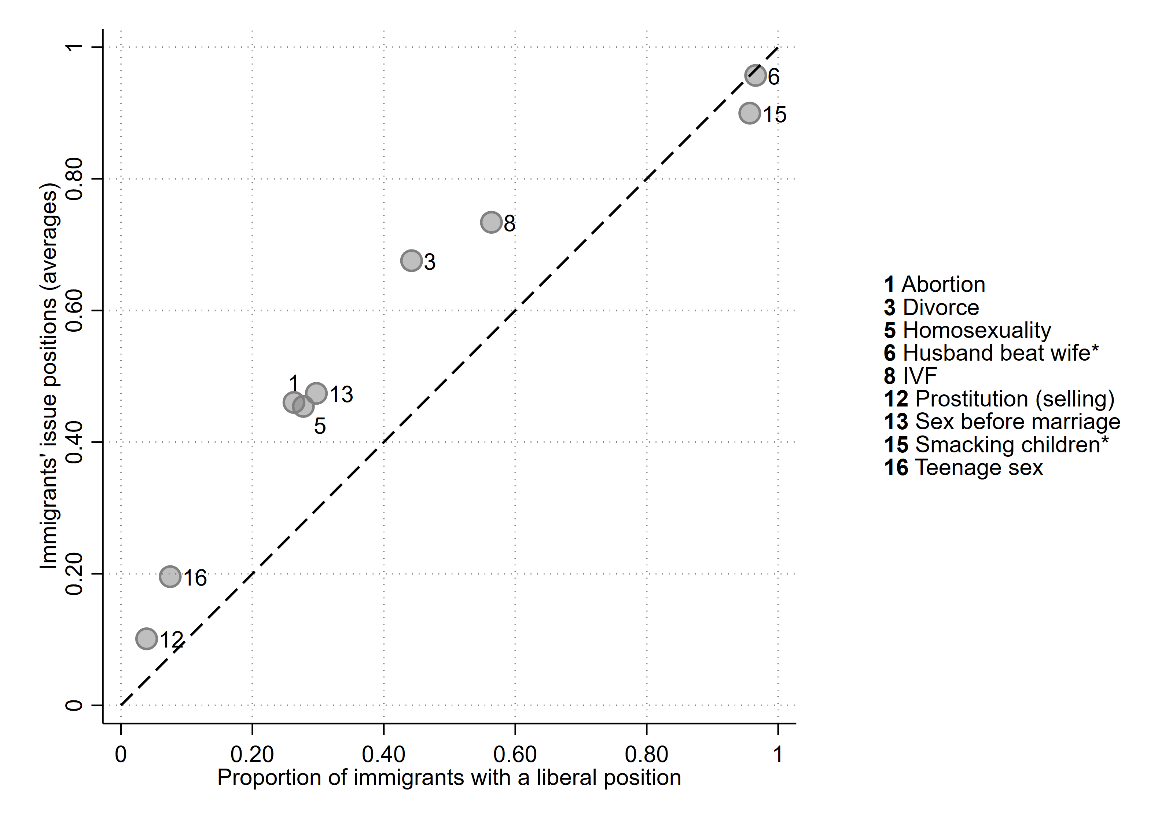

Supplement: Supplementary file 1 — Supplementary Material 1. [file 12978_2025_2161_MOESM1_ESM.docx]
